# Abuse of Vulnerable Older Adults by Designated Surrogate Decision Makers

**DOI:** 10.1093/geroni/igaa057.2467

**Published:** 2020-12-16

**Authors:** Cory Bolkan, Pamela Teaster, Holly Ramsey-Klawsnik, Kenneth Gerow

**Affiliations:** 1 Washington State University, Vancouver, Washington, United States; 2 Virginia Tech, BLACKSBURG, Virginia, United States; 3 Self-Employed, Canton, Massachusetts, United States; 4 University of Wyoming, laramie, Wyoming, United States

## Abstract

Abuse perpetrated by designated surrogates has become highly visible nationally, yet no reliable data exist on its nature or extent. Because vulnerable older adults needing surrogate decision makers typically rely upon others for care, they may be unable to advocate for themselves and are susceptible to abuse. We prospectively gathered Adult Protective Services (APS) data from six geographically diverse counties on over 400 substantiated cases of abuse by perpetrators (53% non-surrogates; 47% documented or claimed surrogate) of vulnerable adults 65+ living in community settings. Most perpetrators (85%) were designated power of attorney, while approximately 8% were guardians, and 7% were representative payees; most perpetrators were family members. Polyabuse occurred frequently. Almost 25% of cases involved a prior substantiated APS report. This presentation highlights how surrogates perpetuate abuse and outcomes on older adult victims. Our findings inform practice and policy for better prevention, detection, investigation, and intervention in these challenging cases.

